# Digitalization of Activities of Daily Living and Its Influence on Social Participation for Community-Dwelling Older Adults: A Scoping Review

**DOI:** 10.3390/healthcare12050504

**Published:** 2024-02-20

**Authors:** Cristina Mendoza-Holgado, Inmaculada García-González, Fidel López-Espuela

**Affiliations:** 1 Occupation, Participation, Sustainability and Quality of Life (Ability Research Group), Faculty of Nursing and Occupational Therapy, University of Extremadura, 10003 Cáceres, Spain; cristinamh@unex.es; 2 Health and Social Services Department, Government of Extremadura, 10004 Cáceres, Spain; 3 Metabolic Bone Diseases Research Group, Department of Nursing, Faculty of Nursing and Occupational Therapy, University of Extremadura, 10003 Cáceres, Spain; fidellopez@unex.es

**Keywords:** daily, everyday technology, occupational therapy

## Abstract

Everyday technology (ET) has been defined as the broad set of devices and artefacts that are currently present in people’s lives both inside and outside of the home. A subgroup within ET is known as everyday information and communication technologies (EICTs). The main characteristic of these technologies is that they can facilitate or disrupt the engagement of individuals in their daily activities. This scoping review aims to identify what is known about how ET can function as facilitators or barriers to occupations, such as the social participation of older adults. The proposed scoping review was conducted in accordance with the Joanna Briggs Institute (JBI) methodology for scoping reviews. The review followed the Preferred Reporting Items for Systematic Reviews and Meta-Analyses extension for Scoping Review (PRISMA-ScR) guidelines and checklist. The scoping review was conducted across five online databases (PubMed, Scopus, Web of Science, MEDLINE and PsycoINFO [EBSCO]) to identify published, peer-reviewed records. Studies were screened by two independent reviewers against the inclusion criteria. This review considered studies concerning the use of ET or EICTs in community-dwelling adults aged over 60 years, with or without cognitive impairment or dementia. All of the articles were in English, and reviews were not included. Eleven papers were selected and showed that despite the increasing demand for technologies of daily life and the digitalization of society and processes, according to our research, few studies addressed the limitations in the social participation of older adults. In conclusion, ET can provide a way to promote and maintain the personal autonomy for older adults in community dwellings. However, cognitive impairment hinders the use of electronic technologies and increases perceived problems.

## 1. Introduction

In Spain, the proportion of elderly individuals has increased to 20.08%. By 2037, the proportion of elderly individuals is estimated to increase to 26.0%. Another indicator known as life expectancy is expected to increase to 85.8 years for men and 90.0 years for women in 2069. Moreover, ageing is more pronounced in rural areas. In general, depopulation processes have been triggered in Spain as a result of high rates of migration from these areas to the expanding urban areas. The extinction of older generations will accelerate this depopulation process in the coming decades [1]. According to the World Health Organization (WHO), between 2015 and 2050, the proportion of the global population aged over 60 is set to nearly double, rising from 12% to 22%. Thus, all countries face major challenges to ensure that their health and social systems are ready to make the most of this demographic shift. This increase emphasizes the importance of appropriate economic, social and health policies to address this situation, and it is necessary to implement a sustainable social model that contributes to promoting personal independence and self-care for as long as possible, thus delaying institutionalization [2].

“Everyday technology” (ET) has been defined in previous studies as being the large set of devices and appliances that are currently present in the lives of people both at home and in the public space. Therefore, ETs refer to common domestic technologies and those technologies that appear in the space outside of a person’s home that all citizens have access to (for instance, ATMs) [3].

One of the main characteristics of ET is that it can facilitate or hinder people’s engagement in their daily occupations. The introduction of new digital devices in the home or in society to perform activities of daily living will require the development of new attitudes and skills in society as a whole [4]. Occupational engagement includes accomplishment in the selected occupation resulting from the dynamic transaction among the client, their context and the occupation [5].

Everyday information and communication technologies (EITCs) are a subgroup of ET. Information and communication technologies include telephones, smartphones, tablets and computers/laptops which enable the storage and exchange of information. In addition to the devices, EITCs include various functions, such as calling, searching for information, sending e-mails and text messages and managing bank accounts. Currently, many activities of daily living necessitate the use of electronic devices such as smartphones and laptops [6,7].

This growth is also reflected in homes, wherein the use of electronic devices has also increased, including functions ranging from basic household appliances to more complex equipment [6]. The outbreak of the coronavirus pandemic during 2020 prompted health authorities to impose the decision for the population to stay at home to avoid contagion first and then to maintain social distance. Isolation has increased the use of new communication models such as voice calls, instant messaging and telemedicine. However, it also brought to light the inequality of access to these communication systems in different groups of the population, an issue known as the digital divide.

There is no doubt that this technological development is potentially beneficial and has great advantages; however, from the point of view of occupational therapy, we have to maintain a critical approach to the consequences that underline this change in the model.

Disadvantages have been identified in the widespread use of technology in different fields, such as education and health, as well as in social groups. Some of the factors that appear to have an influence may be associated with gender, age, education, socioeconomic background or household composition [8]. Technology that is used in an enforced manner and without an alternative (instead of being used to facilitate personal autonomy and independence) would indirectly become a way of limiting social participation and represent an interference of rights [9].

Within the population groups that have been affected by this digitalization, the existing digital gap in the older adult population is remarkable; in addition, even within the same group, differences have been identified between those who suffer from a cognitive impairment and who live in rural areas, among other factors [10,11]. Mild cognitive impairment (MCI) represents a transitional state between healthy aging and very mild Alzheimer’s disease (AD), involving a cognitive decline which is greater than expected for that age [12]. It is characterized by the preservation of functional independence, but minor failures appear in complex activities, such as the use of technology or money management.

The World Health Organization has established social participation as being a social determinant of health as a non-medical factor that influences health outcomes; hence, it represents a modifiable factor. Furthermore, it highlights the need for policies to be oriented towards supporting this participation to achieve universal health coverage [13]. Occupational therapists are professionals who are trained to address barriers to participation. They should work to facilitate participation in everyday life and support participation in meaningful activities to pursue wellbeing as a human right while also avoiding social exclusion [3,14,15]. Participation in cognitive and social activities is associated with health benefits that can prevent cognitive decline among older people at risk of developing dementia [16].

Therefore, occupational therapists must work in accordance with their fundamental precepts, one of which is to implement the necessary actions to encourage and promote occupational justice for individuals or groups [5,17]. The inability to participate fully in community life and meaningful occupations as a result of a lack of accessibility to ET can be a form of occupational deprivation. Furthermore, as occupational therapists, we should consider whether the social construction of older adults in current society is real or whether the issue is being addressed from a paternalistic perspective. This could indicate that the social construction of older people is marked by a set of prejudices and stereotypes that promote so-called ageism [18].

Social participation was included as one of the occupations and is defined as activities that involve social interaction with others, including family, friends, peers and community members and that support social interdependence (community participation, family participation, etc.) [5]. Moreover, in the context of growing population ageing, technologies aimed at helping people age in place play a fundamental role, not only for social participation [19]. In the context of occupational therapy, this is a broad construct defined as the environmental and personal factors specific to each person, group or population that influences engagement and participation in occupations. Technology is indeed integrated into the environment, so how it interacts with people should be a concern of occupational therapy [5].

A determinant of increased participation is an accessible community that has previously been considered from a physical viewpoint. In recent years, the possibility of people with physical disabilities participating in the community has increased as a result of conscious policies such as accessibility legislation [3]. These standards have essentially focused on motor and perceptual difficulties, with priority given to the removal of physical barriers. At present, accessibility should be an inclusive concept that encompasses more than just physical aspects. In the scientific literature, terms such as “gerontotechnology” have been identified to refer to the relationship between ageing and technology, with the aim of contributing to the reduction in the problems of older people during ageing [20].

A preliminary search of JBI Evidence Synthesis, the Cochrane Database of Systematic Reviews and PROSPERO was conducted and revealed no current or in-progress scoping review or systematic review on the proposed topic. The lack of knowledge about how ET can act as a facilitator or barrier in social participation highlights a gap in the literature regarding social participation in older adults.

Three systematic reviews registered in PROSPERO on similar topics to this proposed review were identified [21,22,23]. All of them were focused on people with cognitive disabilities, dementia or mild cognitive impairment. Moreover, one review focused on an assisted living facility and one on assessments for Instrumental Activities of Daily Living.

The aim of this scoping review was to assess the extent of the literature on how technology is used in everyday occupations within the scope of occupational therapy for community-dwelling older adults aged 60 years old or over, with or without cognitive impairment. Hence, this review will identify barriers and facilitators for the use of ETs as well as how they affect occupations, in particular, social participation.

## 2. Materials and Methods

The proposed scoping review was conducted in accordance with the JBI methodology for scoping reviews [24,25,26]. The review followed the Preferred Reporting Items for Systematic Reviews and Meta-Analyses extension for Scoping Review (PRISMA-ScR) checklist [27]. The protocol was registered in the Open Science Framework https://doi.org/10.17605/OSF.IO/YTA4Z (accessed on 10 January 2023).

**Figure 1:**
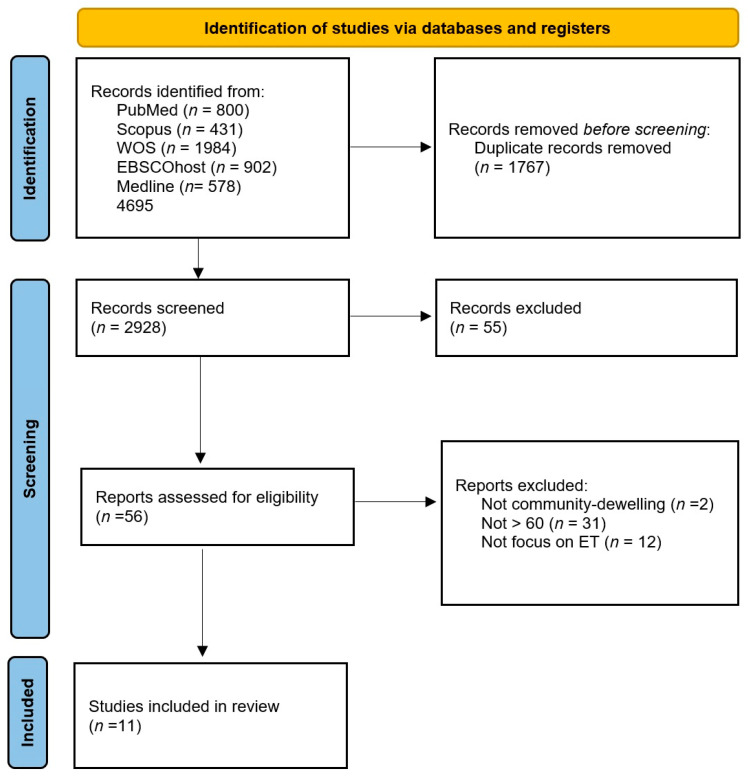
Flow chart.

This research method is beneficial to initiating a research process when the topic is complex and when no previous reviews have been conducted. Moreover, it is helpful to discovering knowledge gaps and to identifying all of the relevant literature. The process is described in a flowchart (Figure 1), which displays the exclusion criteria for why articles were excluded.

The proposed review question was as follows: (1) what studies have been performed on the process of digitalization and the use of technology in older adults within the scope of occupational therapy, and (2) is digitalization a barrier or a facilitator in the social participation of older adults?

### 2.1. Search Strategy

The search strategy aimed to locate both published and unpublished primary studies. The text words contained in the titles and abstracts of relevant articles and the index terms that were used to describe the articles were used to develop a full search strategy for PubMed, which is detailed in Figure 1. The search strategy, including all of the identified keywords and index terms, was adapted for each included database and information source.

Studies published in English were included, without limitations on year of publication. The searched databases were PubMed, SCOPUS, Web of Science, EBSCOHost (PsychINFO) and MEDLINE. Potential reference lists of selected articles were screened for additional papers. An expert librarian helped to construct a search strategy.

### 2.2. Eligibility Criteria

#### 2.2.1. Participants

This review considered papers that included individuals aged 60 years or older who lived in a community dwelling. There were no restrictions of participants due to health conditions. Healthy people or those with a diagnosed illness or pathology (such as mild cognitive impairment, dementia or any physical and mental disorder) were eligible. When the range of age was not evident, we considered eligible individuals if the mean age was over 60 years.

#### 2.2.2. Concepts

This scoping review focused on studies that reported results on ET and the digitalization of activities of daily living as well as their influence on social participation for community-dwelling older individuals. For the purpose of this review, barriers and facilitators were explored. The scoping review was focused on the concept of ET and a subgroup of EITC. Activities of daily living and social participation were considered to be occupations according to the theoretical basics of occupational therapy.

#### 2.2.3. Contexts

This scoping review considered studies focusing on community-dwelling older adults living in a private home. Any papers that included a population living in a nursing home, assisted living facilities or supervised house, among other locations, were not considered for inclusion. Nevertheless, studies with subjects who lived in their own homes but who had a person to assist them on a daily basis were considered to be eligible. There were no restrictions as to whether the population was rural or urban or if it was enrolled in any type of diary center.

#### 2.2.4. Types of Sources

This scoping review considered both experimental and quasiexperimental study designs, including randomized controlled trials, nonrandomized controlled trials, before-and-after studies and interrupted time-series studies. In addition, analytical observational studies including prospective and retrospective cohort studies, case–control studies and analytical cross-sectional studies were considered for inclusion. This review also considered descriptive observational study designs, including case series, individual case reports and descriptive cross-sectional studies, for inclusion.

Qualitative studies were also considered that focused on qualitative data, including (but not limited to) phenomenology, grounded theory, ethnography, qualitative description, action research and feminist research designs.

Finally, neither systematic reviews nor any type of review was considered for inclusion. Text and opinion papers were also not considered for inclusion in this scoping review.

### 2.3. Study Selection, Data Extraction and Synthesis

Following the search, all of the identified citations were collated and uploaded into the bibliographic software Zotero 6.0.18 (Virginia, USA; 9 November 2022), and duplicates were removed. Afterwards, the studies were exported to Parsifal, which is an online tool designed to support researchers in performing systematic literature reviews. Finally, an Excel sheet was used for data extraction.

Data were extracted from papers that were included in the scoping review by two independent reviewers using a data extraction sheet constructed by two authors. One author extracted the data from the included studies into an Excel file. The data extraction included specific details about the paper (such as author and year), study methods and tools, population, concept, context and key findings that were relevant to the review question(s). Any disagreements that arose between the reviewers were resolved via discussion.

## 3. Results

**Figure 2:**
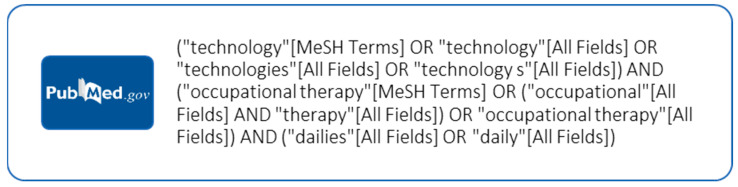
PubMed search strategy.

As illustrated in the PRISMA-ScR flowchart in Figure 1, a total of 4695 sources were identified from searches of five electronic databases. No additional studies were identified via a manual search of the bibliographies. After removing duplicates, we obtained 2928 papers. Based on the title and the abstract, 2772 studies were excluded, and a total of 56 sources remained for full-text screening. Of these, 45 were excluded for the following reasons: 12 studies did not examine ET as we currently consider it, 31 studies included people older than 60 years old and 2 studies included people who were not living in the community. Thus, 11 studies published between 2005 and 2022 met the inclusion criteria for this scoping review. An overview of the selected studies is presented in Figure 2 (flowchart) and summarized in Table 1 (general population) and Table 2 (studies about populations with MCI or dementia).

### 3.1. Population

The examined population mainly consisted of older adults/community-dwelling older adults from urban areas [28,29,30,37], and only Fisch et al. (2020) focused on older people in rural communities [32]. Two studies included a sample of people with MCI [36], and three studies considered a mix of diagnoses [33,34,35] and compared groups of cognitively healthy older adults with MCI and dementia.

The numbers of the evaluated sample were considerably varied, and the range of variation ranged from the smallest sample with six participants [36] to the largest, reaching a total population of two hundred and seventy-four individuals [30]. Most studies were conducted in Sweden (*n* = 6, 55%) [30,32,34,35,36,37], and other studies were conducted in the United States (*n* = 4, 36%) [28,29,31,38] and Belgium (*n* = 1, 9%) [33]. For most studies, the first author was from the field of occupational therapy or occupational therapy departments from several universities (*n* = 8) [28,29,31,32,34,36,37,38], followed by neuropsychologists or departments of psychiatry and neuropsychology (*n* = 2) [33,35] and departments of geriatrics (*n* = 1) [30].

### 3.2. Methodology and Tools

Six studies used quantitative research methodologies [28,29,31,33,34,35], whereas four adopted qualitative research methodologies [32,36,37,38]. Only one study used mixed methodologies [30]. In quantitative studies, the most common data collection assessment was the “Everyday Technology Use Questionnaire” (ETUQ), which was used both in normal [28,30,31] and reduced versions (short version of the Everyday Technology Use Questionnaire, S-ETUQ) [34,35,37]. In qualitative studies, the most prominent data collection methods that were adopted were interviews, explorative interviews, in-depth interviews and focus group interviews.

In addition, some studies were complemented with other standardized screening tools, such as the Montreal Cognitive Assessment (MoCA) [28,31], Mini Mental State Examination (MMSE) [30,33], Cambridge Cognitive Examination (CAMCOG) [33] and memory items, to assess cognitive status. The Frenchay Activities Index (FAI) [28,37] was used to explore instrumental activities of daily living. The Cognitive Disability Index (a-ADL-CDI) was used to measure activities of daily living limited by cognitive impairment [33]. The Assessment of Motor and Process Skills (AMPS) [31,34] and the Management of Everyday Technology Assessment (META) [35] were established for the observation of performance in the use of technologies relevant to the individual.

### 3.3. Relevance and Self-Perceived Ability to Use Everyday Technologies

Several studies have evaluated the relationship between the self-perceived ability to use relevant everyday technologies (as assessed by ETUQ or S-ETUQ) in cognitively healthy older people as compared to those with MCI or AD [28,30,31,34,35,37], whereby they investigated associations between activity engagement (AE), number of available and relevant everyday technologies, usability and cognitive status among older adults [28]. Six studies included older adults with MCI [33,34,35,36,37,38] (Table 3); additionally, in four studies, cognitive status was evaluated by using specific tools [28,30,31,33]. The aim was to determine the relationships between ET and the measured cognitive status. Furthermore, two studies assessed cognitive status and included the AMPS and its interaction with cognitive impairment.

In the study by Walsh et al., associations among activity engagement, the number of available and relevant everyday technologies, ability to use and cognitive status among older adults were investigated [28]. Their results demonstrated that participants with medium and high scores in the FAI reported a greater number of available and relevant ETs. Additionally, Bartels identified that participants with dementia showed a tendency to need more assistance with using the electronic technologies or stopped using them even though they were relevant [35]. Among the findings of the subsequent study by Walsh, it is noteworthy that the number of available and relevant technologies used became the most significant predictor of minimal or moderate needed assistance [31].

Eek investigated the benefits or perceived problems of ET in the homes of 86-year-olds. Their investigation demonstrated, among their findings, that cognitive impairment hindered the use of electronic technologies and increased perceived problems [30]. Another study reported a total of 134 perceived problems with the usage of ET. The most common issues were operating difficulties and problems due to visual and/or hearing impairments. This research also reported that some individuals without a computer considered their lack of Internet access to be problematic [29]. Bartels also referenced differences in self-perceived ability between people with dementia and MCI [35].

Moreover, other authors analyzed the experiences of individuals with MCI participating in out-of-home activities. Participants reported a constant sense of change in their activities and expressed a feeling that some things that "make life easier" for others had an opposite effect on them. The decision to leave or move was also discussed [38]. On the other hand, other researchers explored the possibility of determining indicators of functional decline through the measurement of cognitive status and performance level. Cognitive status was identified as a significant predictor of the need for minimal or moderate assistance [31]. Vermeersch et al. found associations between cognitive decline indices and the performance of more complex activities of daily living (ET, driving and performing complex economic activities)., The authors compared three groups: cognitively healthy elderly, MCI and AD [33].

### 3.4. Engagement and Occupational Performance

To determine how technology influences patterns of participation, some articles focused on specific devices (television, Internet and telephones) [38]. Other authors considered aspects such as the importance of occupational purpose in influencing the incorporation of a new technology into occupational patterns [37,38]. Two studies explored the potential for achieving a satisfactory relationship between occupation and technology in older people [36,38]

Determining how technology influences patterns of participation was one of the goals of qualitative research conducted by Heatwole. Observations and accounts of everyday lived experiences of older people showed the significant role of technology in daily life in shaping patterns of participation [38]. One study addressed the matching of occupational purposes and technology. This study considered that the importance of the occupational purpose influences the incorporation of a new technology into the occupation; if the technology’s utility matched with a highly relevant occupational purpose, it was more likely to be used than if it matched a less relevant purpose. By contrast, the study also pointed out that the purposes of an occupation could change as a consequence of new technology becoming a part of it [37]. However, people with MCI tended to choose information and communication technologies (smartphones, computers, etc.) while people with dementia more often chose household technologies (power tools, coffee makers) [35]. The results show that the most common reason for telephone use and Internet access is the need for social contact [29,30].

Two studies explored the means of achieving a satisfactory occupation–technology relationship in older adults [36,38]. Older adults with MCI presented four different modes in the context of their out-of-home activities and participation in the community: enabling being present, facilitating participation, impeding goals and constraining options; these modes may overlap and change over time. The technology–condition interaction can be mapped across the four modes of participants’ experience, having both enhancing and limiting influences in the context of daily life [38]. Hedman et al. summarized the need for the older person to engage in a continuous process of “downsizing, retaining and updating” to make effective use of technologies in daily life [36].

### 3.5. Population with Mild Cognitive Impairment/Dementia

Six studies included older adults with MCI. The studies presented represent the use of technology in older adults with cognitive impairment from different approaches. In total, the studies address a population of *n* = 130 MCI/SCI and *n* = 130 people with AD/mild-stage dementia. The use of technology in people with MCI is a challenge [34,38] and emphasizes the need to facilitate its use [37]. A few studies highlight the value of assessing advanced technology-related activities of daily living for early detection of cognitive impairment [27,28]. On this topic, Bartels et al. emphasize the benefits of combining the S-ETUQ and the META to gain insight into the individual’s situation. The findings of this study suggest that the ability of older adults with cognitive impairments to use ETs can be depicted with self-perceived reports as well as with observations [35].

### 3.6. Occupational Therapy and Everyday Technology

**Figure 3:**
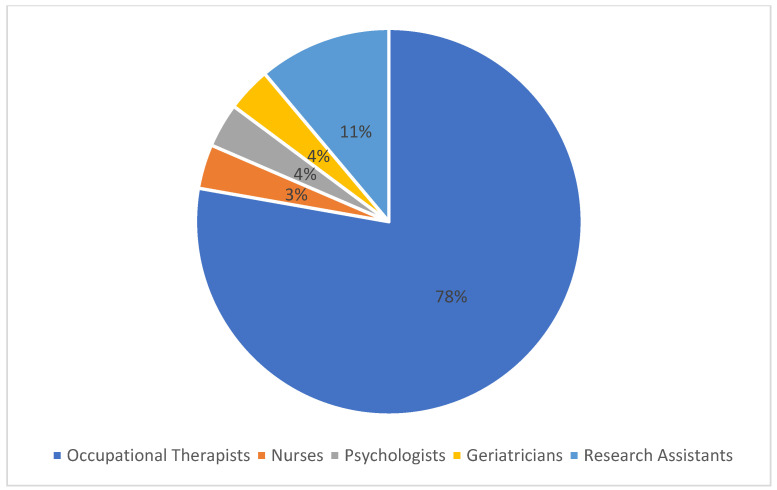
Health professionals involved.

Five studies were published in the *Occupational Therapy Journal* [28,29,33,34,37]; most of the other articles were published in journals related to gerontology and aging. All the main authors or corresponding authors were occupational therapists or members of an occupational therapy department. Some studies [28,29,31,32,33,34,35,37] indicated the persons responsible for conducting the interviews or evaluations for data collection (Figure 3). In total, 27 different interviewers were allocated, and 21 of them were OTs (78%). Regarding the technologies evaluated, the most common were those identified in the ETUQ [28,30,31,33,34,35,37] and divided into seven different areas: (1) home maintenance, (2) information and communication, (3) self-care, (4) maintenance and repair, (5) accessibility, (6) finances and purchasing and (7) travel.

## 4. Discussion

In this scoping review, we aimed to identify what is known about how ET influences facilitators or barriers to occupations, such as the social participation of older adults. Only 11 studies were found that investigated the relationship between technology use and performance in everyday occupations, in particular, social participation in older adults, according to our search strategy.

An interesting aspect of the literature is the variety of concepts found in relation to the results. For instance, “social participation” has been largely used in occupational therapy [5]. However, the lack of consistent use of research terminology related to everyday occupation and social participation is evident in the articles retrieved for this study; our review demonstrated that only three studies mentioned “social participation” literally [30,32,38], whereas other research referred to more general concepts such as “occupational engagement” [37], “activity involvement” [39,40] or “activity engagement” [28] to establish the relationship between the use and performance of ETs and occupational performance in activities of daily living. 

One assumption extracted from the review is that if the utility of the technology coincided with a highly relevant occupational purpose, it was more likely to be used than if it coincided with a less relevant purpose [33]. Conversely, it also indicated that the purposes of an occupation could change as a consequence of the incorporation of a new technology.

Another aspect that is highlighted from our review is that the most common barriers and facilitators have been associated with functional decline. The amount of functional disability due to cognitive, physical or sensory decline is associated with the engagement and use of daily living technology. Regarding engagement, the highest levels of participation in ET use have been correlated with healthy individuals [28]. In particular, many studies have focused attention on the influence of cognitive status and the ability to use technology [28,33,34,35,36,37,38]. One piece of research demonstrated (through performance observation) that people with MCI tended to choose information and communication technologies (smartphones, computers, etc.), whereas people with dementia were more likely to choose domestic technologies (such as power tools and coffee machines) [35]. If a technology meets the control and security needs of the participants in an occupation, it facilitates its incorporation and use [37].

In this regard, some previous review studies have investigated the influence of the use of ET as a facilitator to improve everyday occupations and participation in older persons with mild dementia, mild cognitive impairment or dementia and have included the perspective of health professionals. [41,42]. Additionally, some research has highlighted the role that ET (as a specific activity within instrumental activities) can provide as an early marker to detect the onset of cognitive impairment [33]. This finding is consistent with previous studies conducted by Nygard that reported that people who develop dementia appear to experience subtle changes in complex instrumental ADLs long before the onset of the disease [43].

To delve into the challenges involving technology utilization among individuals experiencing cognitive decline within the framework of aging compared to people experiencing normal aging, certain studies underscore that the engagement with technology is seen as a frustration and barrier for older adults with MCI [38]. Conversely, the growing gerotechnology industry capitalizes on the image of technological innovations as the solution to many of the common challenges associated with ageing in place. So, technologies aimed at helping people age in place play a fundamental role [19].

This scoping review also provides a general overview of the interaction between the presence and relevance of technology with respect to perceived ability. According to ETUQ instruction a technology is not relevant when it is not present in the person’s environment/life or it has never been used and is not intended to used.

Our results demonstrated that several of the articles that we identified include evaluation tools such as the ETUQ, S-ETUQ or META, which investigate the aspects indicated previously. These findings are in conjunction with the assumption that it is important for clinicians to have valid assessment instruments that can provide information on perceived difficulties for older people [31].

In this regard, we can determine a topic for discussion regarding the role of health professionals (in particular, the occupational therapist) in relation to ET. Thus, some articles highlight the involvement of health care professionals in screening for ET use to reduce the overall care that is needed [31]. Furthermore, another article highlighted that interventions aimed at facilitating technology use among older adults should not solely target individuals who experience challenges in using technology [37]. Intervention and support across older adults (who experience challenges in using technology or not) can support occupational engagement and increased participation in society [29,30].

The findings offered by Ryd’s study [37] provide a deepened understanding of occupations by illustrating how a change in the environment, such as a new technological object, can have extensive effects on the dynamics of an occupation. Moreover, through technology, occupational therapists can contribute to promoting functional independence, not only as ET but also as an assistive technology [19]. Hence, the occupational therapy theoretical framework is used to understand how a person’s occupations change and adapt in response to specific conditions or requirements. Regarding community-dwelling older adults, considering everyday technology may inform the fit between a person and their environment and guide intervention to preserve and increase independence and participation. New technological objects can have extensive influence on the dynamics of an occupation [31,37].

This scoping review describes how the experiences and performance in social participation of older adults are altered by the use of new technologies. In line with this statement, another study reports that occupational therapists need to address the area of ET to facilitate occupational performance and increase participation in society [30].

To consider this change in occupational performance in older adults, some research has focused on a range of technologies in general, whereas others have focused on a particular technology [29]. Most of the studies refer to the set of technologies of everyday life described in the ETUQ. However, some are specific to a particular technology, as in the research proposed by Mann et al., which focused on the traditional telephone [29], or from Fischl (2020), which focused on digital technologies (laptops, tablets, printers, mobile telephones, etc.) [32]. Finally, another study focused on everyday technologies related to the performance of some advanced activities of daily living (ET, driving and performing complex economic activities) [33].

Moreover, some papers have addressed the different requirements of people living in rural and urban areas [28,32]. In this manner, qualitative research highlighted the interrelationship between the loss of traditional social support networks in favor of expanding digital opportunities for social participation. Older people recognized increasing possibilities for using digital technologies in everyday life, although they also demonstrated reduced opportunities for social interaction [32].

The limitations of the study were mainly terminological difficulties. First, with regard to technology, before the establishment of the ET concept, many of these technological elements were assimilated as Assistive Technology. Second, the variability of terms to describe occupational "engagement" contributes to the dissemination of data and contributes to the difficulty of identifying the concept of social participation. The subject presented includes a very wide range of topics, so future lines of research should propose systematic reviews of specific questions such as the use of technology for people with cognitive impairment and without cognitive impairment and social participation due the importance of cognitive status and the different needs compared to healthy people.

## 5. Conclusions

Despite the increasing demand for ET and the digitization of society and processes, according to our research, few studies have addressed the limitations in social participation of older adults, although the first studies on this topic were conducted more than 20 years ago. However, there seems to be an imbalance between the studies reported from different countries, with most of the studies emerging from Nordic countries, even though the EU ageing rate indicates progressive ageing in all countries, especially in countries such as Spain. Many of the targeted research studies are focused on the influence of functional and cognitive impairment and its influence on the ability to use technology. 

ET can provide a way to promote and maintain the personal autonomy for older adults in community dwellings. However, cognitive impairment hinders the use of electronic technologies and increases perceived problems. Moreover, ET can be used as a tool for early detection of functional failures in complex activities that may be a symptom of the onset of cognitive impairment. Facilitating access to technology and providing training in its usage, along with the need for alternative or compensatory measures in cases where its use causes frustration, promote the social participation of older adults.

## Figures and Tables

**Table 1:** Characteristics of included articles on the general population.

Study (Year), Country	Aim	Population	Methodology/Tool	Technology Type	Results	Conclusions
Walsh et al. (2018), USA [28]	Investigate associations among activity engagement (AE), number of available and relevant everyday technologies, ability to use everyday technologies and cognitive status.	*n* = 110 adults in an urban area	Observational study: Everyday Technology Use Questionnaire (ETUQ), Frenchay Activities Index (FAI), the Montreal Cognitive Assessment (MoCA).	ET (88 items): Instrumental activities of daily living as well as in social and community activities.	The number of available and relevant everyday technologies was significantly different (*p* < 0.001) among groups that varied in level of AE. The ability to use ETs did not significantly differ among groups. Cognitive status did not explain level of AE when the number of available and relevant ETs was considered.	Increasing the accessibility of available and relevant ETs among older adults in an urban area may increase activity engagement.
Mann et al. (2005), USA [29]	Explore the needs and barriers of the use of the telephone and its features from the perspective of older adults.	*n* = 609	Observational study: questionnaire that included yes or no, multiple-choice, open-ended and Likert scale questions and answers.	Traditional (telephone)	The most common reasons for using a telephone were social contact (98.2%), setting up medical appointments (90.3%), refilling prescriptions (81.2%), business (55.6%) and calling for help/assistance (49.0%).	Therapists should be prepared to address issues related to telephone placement and wiring, furniture placement and provision of information about cost and telephone features that address specific impairments. Therapists can also address issues about background noise, telephone maintenance, nuisance calls and special services
Eek and Wressle (2011), Sweden [30]	Investigate the presence of ET use in the homes of 86-year-old people in Sweden as well as benefits or perceived problems and needs for other technology. Another aim was to study users ‘perceptions of technical development and its influence on their lives.	Total *n* = 274	Quantitative and qualitative design: Mini Mental State Examination (MMSE), ETUQ	Everyday technologies	Watching TV was important. This medium, combined with reading newspapers, was important for obtaining news. The most common problems in the use of ETs were related to visual or hearing impairments or operating difficulties. Regarding access to a computer, cognitive impairment impeded ET use and increased perceived problems.	Occupational therapists need to address the area of ET to facilitate occupational performance and increased participation in society.
Walsh et al. (2020), USA [31]	Investigate whether assessing use of ETs enhanced predictions of overall needed assistance among urban older adults.	*n* = 108	Quantitative, cross-sectional study: ETUQ, MOCA, the Assessment of Motor and Process Skills (AMPS)	Everyday technologies	Cognitive status was a significant predictor of minimal or moderate needed assistance. The number of available and relevant technologies that were used became the most significant predictor of minimal or moderate needed assistance.	As urban older adults must increasingly use everyday technologies to engage in valued activities, health professionals play a role in screening ET use to reduce overall needed assistance.
Fischl et al. (2020), Sweden [32]	Explore older adults’ perceptions about contexts surrounding their social participation in a digital society.	*n* = 18 older adults from rural communities	Qualitative design: Focus group interviews	Access to and use of digital technologies (laptop computers, printers, smart telephones, tablets)	The authors found a juxtaposition between offline social networks and the expanded opportunities that digitalization brings for social participation. Participants revealed reduced opportunities for social interaction, which they ascribed to prioritizing home activities, dwindling social groups and increasing computerized access to services. Concomitantly, participants acknowledged increasing possibilities to use digital technologies in daily life.	Cocreating usable digitalized services and facilitating satisfactory use of digital technologies could support older adults’ social participation through activities that they find relevant in their lives; subsequently, this may enable them to live at home for longer.

AE: activity engagement; ETUQ: Everyday Technology Use Questionnaire; FAI: Frenchay Activities Index; ET: everyday technology; MoCA: Montreal Cognitive Assessment; MMSE: Mini Mental State Examination; AMPS: Assessment of Motor and Process Skills.

**Table 2:** Characteristics of included articles about people with MCI/Alzheimer’s disease.

Study (Year), Country	Aim	Population	Methodology/Tool	Technology Type	Results	Conclusions
Vermeersch et al. (2015), Belgium [33]	Investigate the relationship between functional decline in those three a-ADLs (advanced activities of daily living) and cognitive decline in persons with mild cognitive impairment (MCI), persons with Alzheimer’s disease (AD) and cognitively healthy controls.	45 MCI, 48 AD, 50 cognitively healthy controls	Observational study. MMSE. Cambridge Cognitive Examination (CAMCOG). a-ADL (ET), a-ADL (DRIVING), a-ADL (ECONOMY).	Everyday technology, driving a vehicle, performing complex economic activities	The cognitive disability index for performing complex economic activities and the cognitive disability index for the three advanced activities of daily living domains together differed significantly between the three groups. For the whole sample, the advanced activity of daily living cognitive disability indices correlated strongly with the cognitive measures. Within each separate group, few correlations were found.	The value of assessment of advanced activities of daily living in early cognitive decline is emphasized. Functional impairment in certain a-ADLs can be an early marker for cognitive decline, and evaluation of the performance of complex economic activities can thereby be of great importance.
Ryd et al. (2015), Sweden [34]	Explore associations between ADL performance and perceived ability to use ET among older adults with mild-stage AD and MCI. ADL motor and process ability as well as ability to use ET were also compared between the groups.	AD (*n* = 39) MCI (*n* = 28)	Observational study Short version ETUQ (S-ETUQ) AMPS.	Everyday technology. ADL motor and process performance ability	Significant correlations were found between ADL process ability and ET use in both groups (Rs = 0.44 and 0.32, *p* < 0.05); however, for ADL motor ability and ET use, correlations were found only in the MCI group (Rs = 0.51, *p* < 0.01). The MCI group had significantly higher measures of ADL process ability (*p* < 0.001) and ET use (*p* < 0.05).	ADL performance ability and perceived ability to use ET are important to consider in evaluations of older adults with cognitive impairments. Group differences indicate that measures of ADL performance ability and ET use are sensitive enough to discriminate the MCI group from the AD group with individually overlapping measures.
Bartels et al. (2020), Sweden [35]	Evaluate the relationship between the self-perceived ability to use ET and observable performance of self-chosen and familiar (but challenging) ETs in people with mild cognitive impairment (MCI) or dementia.	Total *n* = 79 MCI (*n* = 41) Dementia (*n* = 38)	Observational study FAI MMSE S-ETUQ (33 items). Management of Everyday Technology Assessment (META).	Everyday technologies	In the dementia group, self-perceived report and observational scores correlated at a medium significance level (Rs¼0.44, p¼0.006). In the MCI group, no significant correlation was found.	Benefits of combining the S-ETUQ and META to gain knowledge about the individual’s situation were found. The findings of this study suggest the ability of older adults with cognitive impairments to use ETs can be depicted with self-perceived reports as well as with observations.
Hedman et al. (2016), Sweden [36]	Explore how persons with mild cognitive impairment relate to technology as a part of and as potential support in everyday life—both present and future.	*n* = 6	Qualitative in-depth interviews.	Everyday technologies	There are three different ways of relating to existing and potential future technology in everyday occupations as a continuum of downsizing, retaining and updating.	Persons with MCI may relate to technology in various ways to meet the needs of downsizing, which simultaneously may involve downsizing, retaining and updating technology use.
Ryd et al. (2018), Sweden [37]	Explore what can drive and hinder the incorporation of ET into occupations and how new technology affects occupational engagement and performance among older adults.	*n* = 11	Explorative interview, semistructured S-ETUQ, FAI.	Everyday technologies	The degree of match of the participants’ perceptions of occupational purposes, the type of technology users they strived to be and their need to feel secure and in control in the occupation was essential for the choice of incorporating a new technology and for satisfaction with the altered occupations.	Occupational engagement and performance in relation to technology use can be facilitated, which is useful knowledge for stakeholders who are developing and implementing new technology as well as those who encounter older adults with the needs or desire to use technology in their daily occupations.
Heatwole Shank (2022), USA [38]	Explore the nature of out-of-home experience with technology in the context of daily life situations for community-dwelling older adults with MCI.	*n* = 10	Qualitative design go-along interview.	Everyday technologies	The individuals in this study regularly engaged in activities in the community, despite experiencing changes in their cognitive abilities. Observations and accounts of quotidian experiences showed the significant (yet sometimes tacit or hidden) role of technology in daily life that shaped these patterns of engagement. Key findings include the four modes of experiencing this technology landscape, whereby technology served to enable, facilitate, impede and outright constrict participation for this population.	In representing the experiences of older adults as technology users, social discourse and media have a tendency towards caricature: technology is seen as a frustration and barrier for older adults. Conversely, the growing gerotechnology industry capitalizes on the image of technological innovations as the solution to many of the common challenges associated with ageing in place.

a-ADLs: advanced activities of daily living; MCI: mild cognitive impairment; AD: Alzheimer’s disease; MMSE: Mini Mental State Examination; CAMCOG: Cambridge Cognitive Examination; S-ETUQ, Short Version Everyday Technology Use Questionnaire; AMPS: Assessment of Motor and Process Skills; META: Management of Everyday Technology Assessment; FAI: Frenchay Activities Index; ET: everyday technology. The following section synthesizes results across the 11 studies and presents findings organized by emerging issues.

**Table 3:** Cognitive status sample characteristics.

Author	Total Sample	Cognitively Healthy Control/ MCI ^1^	MCI ^2^ SCI ^3^	Alzheimer’s Disease/Mild-Stage Dementia
Vermeersch et al. [33]	143	50	45	48
Ryd et al. [34]	67		28	39
Bartels et al. [35]	76		41	38
Hedman et al. [36]	6	1	4	1
Ryd et al. [37]	11	5	2	4
Heatwole et al. [38]	10		10	

^1^ NCI, no known cognitive impairment; ^2^ MCI: mild cognitive impairment; ^3^ SCI: subjective cognitive impairment.

## Data Availability

The datasets generated during and analyzed during the current study are available from the corresponding author upon reasonable request.
